# Uncertainty and probability in neonatal end-of-life decision-making: analysing real-time conversations between healthcare professionals and families of critically ill newborns

**DOI:** 10.1186/s12904-023-01170-z

**Published:** 2023-05-03

**Authors:** Regula Limacher, Jean-Claude Fauchère, Deborah Gubler, Manya Jerina Hendriks

**Affiliations:** 1 Department of Neonatology, University Hospital Zurich, University of Zurich, Zurich, Switzerland; 2 Paediatric Palliative Care, University Children’s Hospital Zurich, University of Zurich, Zurich, Switzerland

**Keywords:** Neonatology, Palliative care, End-of-life, Decision-making, Communication, Uncertainty

## Abstract

**Background:**

A significant number of critically ill neonates face potentially adverse prognoses and outcomes, with some of them fulfilling the criteria for perinatal palliative care. When counselling parents about the critical health condition of their child, neonatal healthcare professionals require extensive skills and competencies in palliative care and communication. Thus, this study aimed to investigate the communication patterns and contents between neonatal healthcare professionals and parents of neonates with life-limiting or life-threatening conditions regarding options such as life-sustaining treatment and palliative care in the decision-making process.

**Methods:**

A qualitative approach to analysing audio-recorded conversations between neonatal team and parents. Eight critically ill neonates and a total of 16 conversations from two Swiss level III neonatal intensive care units were included.

**Results:**

Three main themes were identified: the weight of uncertainty in diagnosis and prognosis, the decision-making process, and palliative care. Uncertainty was observed to impede the discussion about all options of care, including palliative care. Regarding decision-making, neonatologists oftentimes conveyed to parents that this was a shared endeavour. However, parental preferences were not ascertained in the conversations analysed. In most cases, healthcare professionals were leading the discussion and parents expressed their opinion reactively to the information or options received. Only few couples proactively participated in decision-making. The continuation of therapy was often the preferred course of action of the healthcare team and the option of palliative care was not mentioned. However, once the option for palliative care was raised, the parents’ wishes and needs regarding the end-of-life care of their child were obtained, respected, and implemented by the team.

**Conclusion:**

Although shared decision-making was a familiar concept in Swiss neonatal intensive care units, parental involvement in the decision-making process illustrated a somewhat different and complex picture. Strict adherence to the concept of certainty might impede the process of decision-making, thereby not discussing palliation and missing opportunities to include parental values and preferences.

## Background

Despite the increased possibility of sustaining life and thereby often improving outcomes for critically ill neonates [1], children still face the greatest risk of death in their first 28 days of life [2, 3]. Healthcare professionals (HCPs) working in a neonatal intensive care unit (NICU) take care for neonates at the threshold of viability or those who are critically ill and their families, whereas a significant number of critically ill neonates face potentially adverse prognoses.

According to the British Association of Perinatal Medicine (BAPM), a neonate with an identified life-limiting or life-threatening condition fulfils the criteria to receive perinatal palliative care (PPC) [4]. PPC is defined as an active and total approach to care, from the point of diagnosis or recognition, throughout the child’s life, death and beyond and is further described as the holistic management of supportive end-of-life (EOL) care following multidisciplinary agreement on eligibility [4, 5]. PPC follows a family-centred approach and involves the family as an integral member of the healthcare team [6]. The diagnosis of a life-limiting or life-threatening condition of a newborn child [5] often hits affected families unpredictably, creating feelings of anxiety and stress, and calling for support on many levels [4, 7, 8]. Hence, it is of vital importance that HCPs working with neonates with life-limiting or life-threatening conditions and their parents meet the requirements of competent palliative care [4, 9, 10]. However, the current stage of research suggests both variability in practice and a lack of adequate provision of PPC in Switzerland [10–14].

In PPC, both parents and neonatal HCPs are confronted with ethical and decisional challenges. There is (inter)national consensus on shared decision-making (SDM) being the preferred clinical [15–18] and ethical [15, 18] approach to treatment decisions on the care of critically ill infants; this approach is also preferred by the Swiss population [19]. Between the two extremes of an optimal decision and a decision that would cause harm, there is a morally significant gap, called the grey zone of decision-making, where parental authority weighs heavily and SDM becomes important [20, 21]. In neonatal care, the SDM process is considered an intermediary between the extremes on the decision-making spectrum of paternalism versus patient or parental autonomy. It is characterized by a reciprocal exchange of information between HCPs and parents and emphasizes the importance of eliciting parental values, goals, and decision-making preferences, e.g., the desired degree of decisional responsibility or amount and type of information [15]. Making shared treatment decisions has shown to be inherently connected to the communication process and as such enhanced by high-quality and compassionate communication [8, 22].

Hence, neonatal HCPs require a diverse range of communication skills ranging from interdisciplinary communication regarding everyday plans to conversations with families about diagnosis, prognosis, illness severity, decision-making, and goals of care. When counselling parents about the critical health condition of their newborn child, skilful communication has shown to distinguish between successful or unsuccessful encounters and helps to alleviate short- and long-term stress for parents [8, 10, 23, 24]. However, there is little data on how HCPs and parents proceed in such conversations [10, 25–27]. There are retrospective studies describing HCPs perceptions on SDM in Swiss perinatal centres [14, 25], but capturing actual interactions through audio recordings remains lacking. Recently, de Vos et al. (2015) and Shaw et al. (2020) examined ways in which HCPs and parents engage in decision-making and how parental or HCPs initiative in decision-making enables or hinders parental involvement, respectively [26, 27]. Communication research in neonatal EOL care warrants in-depth research given the high burdens placed on families and HCPs and the fact that the majority of all paediatric fatalities occur in the neonatal period.

Therefore, the objective of this study was to prospectively observe the conversation patterns and content between HCPs and parents of neonates with a life-limiting or life-threatening condition. We sought to explore (a) how HCPs and parents communicate during postnatal conversations and (b) to what extent palliative care is addressed. This will allow for an illustration of the current standard of the SDM process and the involvement of palliative care in these complex situations in Swiss perinatal centres.

## METHODS

### Design

This study followed a mixed method research design during data collection and focused on the qualitative analysis of the data to provide an in-depth picture of the communication patterns in the decision-making process. In order to ensure anonymity of individual cases, analysis of minimal quantitative data of the study population was conducted. In this article, we present a qualitative approach to prospectively analysing audio-recorded HCPs-parent conversations, a known method in palliative and EOL care communication research [26, 28, 29]. In addition, the patients’ medical records were reviewed for aspects relating to palliative care services.

### Participants

Criterion-based, purposive sampling was applied for the recruitment of participants. Perinatal centres with level III NICUs in Switzerland [30] were eligible for this study since these centres treat most PPC cases. A level III NICU refers to a highly-specialized tertiary perinatal center composed of a level III obstetrical and neonatal unit, where - in addition to the functionalities of level I and II - all critically ill newborn infants are being referred to, preferably before birth [30]. Conversations were eligible when neonatologists considered the possibility of (1) withholding or withdrawal of intensive care measures, (2) redirection of care from life-sustaining to EOL care and/or (3) palliative treatment options to become a topic in the conversations with the parents in the near future and (4) participants fulfilled the criteria for PPC as defined by the BAPM [4]. Additionally, follow-up conversations pertaining to the cases were included. The sample included all parents and HCPs present during the conversations. Excluded were parents under 18 years of age, parents with limited linguistic or cognitive understanding, or parents who refused study participation.

### Data collection

Data collection was conducted between January 2020 and June 2021. First, two authors (MJH and JCF), a local study investigator and/or a study nurse would check for eligible cases. At times the last author (DG) was consulted to examine eligibility. Second, the neonatal HCP assigned to the family would be approached to review eligibility. Third, upon meeting the eligibility requirements the assigned HCP would approach the family and provide information about the purpose of the study. All persons attending the conversation (i.e. parent(s) and HCPs) obtained written informed consent. For every follow-up conversation oral informed consent was obtained. A total number of nine families from two level III NICUs in Switzerland were recruited to participate in the study. One participant was later excluded from the study because the infant did not meet the eligibility criteria for PPC. A total of eight families with 16 conversations were included in the analysis. The patients’ medical records were extracted from the electronic patient document system. Documentation of notes from three interdisciplinary team meetings were missing in two cases. Confidentiality of data was maintained throughout the study. Access to research data was restricted to investigators and all data was stored as password protected computer files.

Before the conversations, parents received written and oral information about the purpose of the study and were informed that they could refuse or revoke their participation at any time. They were ensured anonymity and confidentiality of the data and provided informed consent.

### Data analysis

Audiotapes were transcribed verbatim and anonymized by research assistants with at minimum bachelor’s degree using the *f4 transkript* software (version 8.1.1). The transcripts were analysed using the *Atlas.ti* software (version 22.0.2) applying a qualitative content analysis following the approach from Kuckartz (2019) [31]. Weekly meetings were held between the first author (RL) and the two last authors (DG and MJH) to provide continuous input to the data analysis. First, three authors (RL, DG, MJH) collaboratively developed a coding guide based on palliative and EOL care communication research [26, 28]. Then, the first author thematically coded two transcripts along the refined coding scheme. Second, two senior authors (DG and MJH) independently reviewed these transcripts and coded additional text segments. The coding scheme was then refined with topics inductively evolving from the transcripts. Third, the first author coded all transcripts, which were discussed and refined further in the weekly meetings. Lastly, the coded segments were systematised and categorised into themes presented in the results after a discussion with all authors. Additionally, the medical records were reviewed and analysed for any documentation related to palliative care. Representative quotes were selected and presented in the text and tables. To ensure accuracy and quality of participants’ quotes, back-to-back translation was conducted [32].

## Results

### Study characteristics

Eight mothers and fathers of neonates participated in the study, which resulted in recording a total of 16 conversations. All participant and conversation characteristics are shown in Table 1. With regard to the neonates, seven were born extremely preterm and one was late preterm. According to the BAPM classification [4], seven neonates were assigned to category four and one to category two (Table 2). Five out of eight neonates died over the course of their stay in the NICU (i.e., 3–31 days of life), one died during hospitalisation on the 287th day of life, and two were discharged to home. All conversations took place after a team meeting, which evaluated the neonate’s situation in an interprofessional manner. All conversations were attended by at least one neonatologist and one neonatal nurse. Furthermore, in two conversations the head of medical genetics or the head of paediatric palliative care participated, respectively. Discussions about decision-making were held by neonatologists, whereas neonatal nurses’ contributions were, if any, mainly related to aspects such as the condition and care of the neonate or the organisation of inpatient services. Conversations lasted approximately 18 to 85 min.

**Table 1:** Basic characteristics of neonates, parents, and conversations

Case	Characteristics of neonates						Characteristics of parents	Characteristics of conversations			
	Gestation week at birth	Weight at birth (Grams)	Twin	Key Diagnosis (Other than prematurity)	BAPM Categorisation	Death (Day of life)	Age mother	Mother tongue	Translator	Nr of consults	Duration (Min)
1	23	500–1000	No	Pulmonary hypoplasia, severe bronchopulmonary dysplasia	3, 4	*	25–29	Yes	No	1	37
2	25	500–1000	No	lntracerebral haemorrhage lV° bilaterally	4	3	35–39	Yes	No	1	22
3	24	500–1000	Yes	IVH III° bilaterally, very extensive venous infarction bilaterally, left panhemispheric and left frontoparietal	4	11	25–29	Yes	No	1	33
4	24	500–1000	No	IVH IV° right and II° left	4	18	30–34	No	Yes	3	28–64
5	24	500–1000	Yes	Severe IVH III° bilaterally with venous infarction left (frontal to temporal)	4	*	35–39	Yes	No	1	85
6	24	500–1000	No	IVH III° bilaterally, Parenchymatous haemorrhage IV° bilaterally (parietal bilaterally, frontal right), Posthaemorrhagic hydrocephalus, Parenchymatous haemorrhage right cerebellum hemisphere	4	6	30–34	Yes	No	1	35
7	24	< 500	No	Volvulus without malrotation, Differential diagnosis necrotizing enterocolitis Bell’s stage IIIa	4	287	30–34	No	Yes	2	33–62
8	36	1000–2000	No	Congenital central hypoventilation syndrome, Clinical suspicion of Hirschspung’s disease	2	31	35–39	Yes	No	6	18–73

* Discharge home

Abbreviations: *IVH* Intraventricular haemorrhage, *n/a* not applicable

**Table 2:** Conditions for Perinatal Palliative Care, adapted from BAPM (2010) [4]

*Category 1*	Ante- or postnatal diagnosis of a condition not compatible with long-term survival, e.g. anencephaly.
*Category 2*	Ante- or postnatal diagnosis of a condition that carries a high risk of significant morbidity or death, e.g. severe renal disease with oligo-/anhydramnion, severe congenital heart disease.
*Category 3*	Babies born at the margins of viability, where intensive care has been deemed inappropriate, e.g. extremely preterm infants with a gestational age of 22 0/7 to 23 6/7.
*Category 4*	Postnatal conditions with a high risk of severe impairment of quality of life *and* when the baby is receiving life support, e.g. severe hypoxic ischemic encephalopathy.
*Category 5*	Postnatal conditions which result in the baby experiencing “unbearable suffering” in the course of their illness or treatment incompatible with survival, e.g. severe necrotizing enterocolitis with extended bowel necrosis.

### Communication in postnatal conversations

Overall, the neonatal team would initiate a consult to inform parents about their child’s medical status and to address the treatment course. If parents mentioned concerns, any questions or misunderstandings were clarified by the HCPs. Conversations between the HCPs and parents included discussions and decisions about life-sustaining interventions and/or EOL care. In this context, three themes emerged from the analysis: (A) the weight of uncertainty in diagnosis and prognosis, (B) the decision-making process, and (C) palliative care.

### A) handling of uncertain diagnosis and prognosis

Three forms of uncertainty were identified in the conversations: diagnostic uncertainty, uncertainty in short-term prognosis, and uncertainty in long-term prognosis (see Table 3).

**Table 3:** Uncertainty

Uncertainty	Illustrative Quotes
Diagnostic uncertainty	**Neonatologist A**: A*nd there the statement of Neonatology [and the paediatric Pneumology and Neuropediatrics] is absolutely clear that the genetic diagnostic confirmation is really needed.* **Neonatologist B**: *Mhm* **Neonatologist A**: *Yes. Because else there is…That is the only diagnostic test that gives us sufficient certainty in a situation like that.* ***(…)*** **Father**: *I see… (sigh) indeed, just when one would assume the situation will not improve, we have now discussed yesterday and the day before, a resuscitation order.* **Neonatologist A**: *Mhm* ***Father***: *And for me it was more or less always clear that I don’t want [the child] to be mechanically resuscitated and today my wife and I have also discussed that we do not wish for intubation or drug resuscitation either, simply because we do not feel it is something [the child] will recover from.* **Neonatologist A**: *Mhm, yes.* **Father**: *So given the underlying medical situation, which we would like to address once more, as often you come so close to the point, where you think, ‘oh my god oh my god’, but we don’t want [the child’s] ribs to be fractured as well.* **Neonatologist A**: *Mhm, yes. I have to say… from our perspective it is really difficult, as long as we do not know what [the child] has, as we are not a 100% certain that it is something [the child] could not improve from, and as long as the diagnosis is not determined, uhm, it’s what we in agreement have discussed, that at the moment from our point of view, we continue in a curative way. That means also to intubate the child if that is deemed necessary. I would not assume it is necessary…but I do know it is…* **Father**: *But if it came to that, it would really distress us* **Neonatologist A**: *Mhm* **Father**: *I have to say*. (Case 8, conversation 1)
Uncertain short-term prognosis	**Neonatologist**: *Since we do not directly have our backs up against the wall, we do not have to say: if one more thing arises, we have no options anymore. And in case of [name of neonate], [the child] has now been treated. We don’t know yet exactly what it is, but [the child] is considerably extensively treated with antibiotic. Here I would also not say we are having our backs up against the wall.* (Case 5)
Uncertain long-term prognosis	**Neonatologist**: *It would be for the time being and to be discussed by us again later. But how we assess it right now, at least on the long-term, is that the chance is still there that [the child] can still develop well, but of course there is also the risk that [the child] does not. However, at the moment for us the risk does not outweigh. Right now, we would not say it is more likely [the child] develops poorly rather than [the child] still has a chance to develop well, despite his condition.* (Case 4, conversation 1)

First, diagnostic uncertainty occurred when genetic or medical tests were pending diagnosis. The neonatal team relied heavily on diagnostic certainty to weigh the different options of life-sustaining and EOL care. In one case, a genetic test was proposed to diagnose congenital central hypoventilation syndrome. In the first conversation, the parents inquired about possible actions if the genetic diagnosis was confirmed and already wished to discuss palliation as an option before having the test results. The neonatologist very clearly stated that the team required a genetic diagnosis before discussing it. While the genetic diagnosis of the congenital central hypoventilation syndrome was ongoing, the neonatologist advocated for further diagnostic testing of the Hirschsprung’s disease, which could considerably aggravate the course of disease.

The second form of uncertainty identified was the uncertain short-term prognosis of the ongoing medical course ranging from clinical improvement to death. This was observed predominantly in cases with cerebral or intestinal diagnoses. In such cases, the life-sustaining treatment was considered by HCPs as long as the child’s medical situation was ambiguous and the short-term outcome highly indeterminate. Moreover, if there were viable life-sustaining therapy options, the neonatal team did not state the therapeutical limits of care.

Finally, the uncertainty in long-term prognosis could be observed to a greater or lesser extent in all cases. To determine the prognosis of long-term physical, functional, cognitive, and/or behavioural impairments, the neonatal team relied heavily on evidence-based literature and the clinical presentation of the neonate. The long-term prognosis always contained a probability for a particular impairment to occur, and thus, a minor and/or major uncertainty always remained. Given major uncertainty in long-term prognosis, the neonatal team predominantly did not discuss EOL care as an option with the parents.

### B) decision-making

All parents and HCPs were at some stage confronted with the decision of whether to continue life-sustaining therapy or pursue EOL care for the corresponding child. Both centres had internal policies, in which SDM was established as the preferred approach for decision-making. In one centre, SDM was described as “the process of interaction between parents, infants, and HCPs in which a mutually elaborated decision is reached based on shared information, whereby the level of involvement may change over time, and the role of those involved needs to be continually assessed and adapted”. Team and parental meetings were mentioned as mandatory for its implementation. In the other centre’s guidelines, a definition of the SDM process was missing; however, SDM was considered to have the highest ethical priority in the context of uncertainty. This meant that parental involvement in decision-making took place in the form of consultations with parents. Possible courses of action were first agreed upon within the neonatal team and then presented as options to the parents in an understandable, non-directive, and step-by-step manner. Furthermore, parental preferences to participate in the decision-making process were documented.

In the conversations analysed, neonatologists often conveyed to parents that they considered decision-making as a “shared venture” and emphasised the importance of accompanying and supporting parents through this process.

#### Parental decision-making preferences

In both internal policies, the assessment of decision-making preferences, i.e., the desired degree of decisional responsibility, was an essential component of SDM. However, only one centre documented parents’ preferences in their interdisciplinary team meetings and in the electronic patient document system, i.e., the neonatal team elicited the extent and willingness of parents to participate in the SDM process. In the other centre, it remained unknown whether, how, and when this information was obtained. In the conversations analysed, it was not observed that neonatal HCPs ascertain parental preferences. One couple actively expressed their preference towards the neonatal team, i.e., they explicitly stated their desire for decision-making support from the neonatal team. In another case, a mother mentioned her preference not to want her child to suffer and be relieved from the pain, however, this was not further addressed by HCPs. The neonatal team did not discuss palliation as an option at this point of time and pursued the path of life-sustaining treatment.

#### A proactive or reactive approach

At some point in the conversations, all parents expressed their values, perceptions, wishes, and/or beliefs. Parents either took a proactive or reactive approach in providing their opinion towards decision-making based on the information they received. Only a few couples proactively participated in decision-making and initiated the discussion on treatment options. For example, after noticing an aggravation of their child’s condition, one couple raised their concerns:

***Father***: *We do see [our child] has difficulties and for us, there is a certain point that where it [diagnosis] gets confirmed, we would say we don’t want to enforce life upon [our child] and keeping [our child] alive only with machines. For us that is very clear. [****Neonatologist***: *Mhm] And that’s why it would be one of the questions for this conversation: What for steps are needed?* (Case 8, Conversation 1)

In most cases, the HCPs were leading the discussion and parents expressed their opinion reactively to the information or options received, which were presented in varying degrees of directiveness or non-directiveness by neonatologists. The following extract illustrates the parents’ reactive approach:

***Neonatologist***: *Above all, there are both possibilities. Either, and despite the situation, we carry on with the therapy, with the likelihoods as I mentioned [****Father***: *Mhm] or, and you have to say ‘we cannot and do not want to imagine that’.****(…)****We do not actively want to end something, but we would not continue the efforts being made. And when we let things go their natural cause, are we clear what… what that would mean. And uhm… we do not want to orchestrate a proposal for you, that is why we [****Father***: *mhm] offer you both options. And of course, our first interest is how you would see this.*

***Father***: *Yes…(sigh) there is somehow again this feeling it does not matter so much how you decide, it is just…you struggle with the decision anyway, regardless of whether you decide for or against it, somehow. [Pause 9 s]*

***Mother***: *For me it does not make any sense this way. (sigh) When I am honest [Pause 9 s]* (Case 3).

#### Timing of parental involvement

Although neonatologists positively expressed their attitude towards taking a “shared path”, parental involvement in the decision-making process illustrated a somewhat different and complex picture. This was related to the timing of parents’ involvement in the decision-making process, which seemed to be driven by the level of uncertainty.

If the neonatal team considered that the threshold of certainty was not yet reached, it was common to use a “wait and see” approach. This meant continuously exchanging and re-evaluating information with the parents in case the situation would worsen. For one child, diagnosed with an intraventricular haemorrhage grade °IV on one and °II on the other side, the neonatologist provided the parents with a long-term prognosis for a moderate to severe neurological impairment. Thereafter, the neonatologist added that the team would continuously reassess and relay their information to the parents. Hence, very often decision-making options were presented to parents in the event that the child’s medical situation deteriorated or when a certain prognosis for a poor outcome could be given. However, in such cases, this often implied that withdrawing care and/or palliation was the only remaining option as is illustrated in the following quote:

***Neonatologist***: *Because this is, here we come, mh, this is a part, this is the part of the brain. [****Father***: *yes] There is the other part that you see on the lung. [****Father***: *yes] Changes that are quite important and happen quickly. That means, mh, that even when [the child] will survive it, in terms of the brain [functionality], nonetheless there will still be long-term intubation and a lot of [****Father***: *yes] support is required afterwards. And…and that’s it, you have to look at the whole picture. [****Father***: *Of course] And there will still be worries. [****Father***: *Yeah] So we have discussed this in our team and we think that it is probably best to ‘redirect’ care. For… forever. I do not know how you feel about that.*

***Father***: *Since last night, and also this morning, we spoke a lot about that. And yes, that is the direction we have to take. When you say ‘redirect’, then ‘redirect’ means to stop intensive care and let go?* (Case 2)

#### Parental reasoning in decision-making

Parents reasoned in various ways when faced with participating in the decision-making for their child. The best interest of the child served as guidance for all parents in the decision-making process. Parents specified not wanting their child to experience pain or suffering. If further medical treatment was perceived to be a high burden, it was often decided to discontinue life-sustaining interventions. Three couples shared their perceptions about what it would mean to raise a child with disabilities. Although these parents had differing views, they each formed a strong opinion on the matter. Further, some couples expressed their thoughts about the worthiness of life and considered these in their decision-making. Parents of neonates with cerebral conditions specifically held an accurate assessment of long-term impairment as important. However, they expressed concern about long-term outcomes if they opted for life-sustaining therapy. Most parents articulated a strong emotion of not wanting to abandon their child. One couple voiced fear regarding making the “wrong decision” when deciding on EOL care and that the child “could perhaps have had a better life” than they considered it to be. By contrast, in one case, the uncertain future, especially with regard to medical long-term complications and the associated high level of care, was a crucial argument for the parents to decide on EOL care.

### C) Palliative Care

Both NICUs involved in this study incorporated internal neonatal palliative care and ethics concepts. Within the records of the preceding team meetings, a discussion about both options, life-sustaining interventions and palliation, were documented.

When life-sustaining therapy had been decided within the neonatal team as the preferred course of action, this was observed to be communicated in different ways. More frequently, the neonatal team did not consider palliation to be a medically and/or ethically justifiable option, subsequently suggesting the continuation of therapy and not presenting the topic of palliation to the parents. In one case, the neonatal team documented there was no sufficient reason to actively propose palliation but they would support a palliative pathway should the parents express such a wish. In another case, the neonatal team did not discuss palliation as an option and pursued the curative path at the point of time when the mother raised the thought of EOL care for her child. The child died of complications in the course of the first year of life.

Ultimately, a total of five neonates received EOL care during their stay in the NICU. In four cases, palliation was agreed upon as the preferred path within the team and options for palliation and life-sustaining interventions were subsequently presented to the parents. The team presented the options in a non-directive way in one case, whereas in the other three cases palliation was suggested rather than presented as an option. In these three cases, parents followed a reactive approach and opted for palliation. Three of these neonates died within less than 24 h after the conversation was held, and one neonate died two days later. In the fifth case, in which the infant was diagnosed with congenital malformation syndrome, the parents followed a proactive approach and raised their wish for palliative care early on. The neonate died on its 31st day of life.

Overall, once the decision for palliative care was made, the time frame for redirection of care was adjusted to the parents’ needs. As such, the parents’ wishes and needs regarding the care of their child were obtained, respected, and implemented. For example, the time needed for the grieving and farewell process were individualized. Likewise, parental concerns and fears were addressed, such as with discomfort, suffering or stress during extubation. Above all and consistently, it was important for all parties that the child would not experience pain or suffering.

## Discussion

Our findings have described the communication between HCPs and parents of neonates with a life-limiting or life-threatening condition in Swiss NICUs from which several implications can be drawn. These relate to the aspect of uncertainty in such complex situations, the practice of SDM, and considerations of palliative care.

Uncertainty has always been part of medicine [33]. Hence, the ability to navigate uncertainty is a prerequisite for effective care [34]. Recent studies have underlined that disclosure and discussion of uncertainty from HCPs can increase parents’ satisfaction and bereavement process [35–38] and are essential for open, respectful, and trustworthy communication [36, 39, 40]. The findings of our study reveal an uncertainty-based communication style by neonatologists which can impede the SDM process with parents of critically ill neonates. In informing parents of possible courses of action, treatment options differing from the life-sustaining approach were often not mentioned until diagnostic certainty or a substantial degree of certainty of a poor prognosis had been reached. In fact, at the time neonates were offered withdrawal of therapy, most had a high likelihood of imminent death. In support of our findings, uncertainty has previously been identified as a barrier to EOL decision-making for neonatologists [41], where higher levels of uncertainty hindered parental involvement in decision-making. Prins et al. (2022) observed that neonatologists often suggest a “wait and see” approach when health conditions deteriorate, whereas practical uncertainties, such as the infant’s dying process, were often only addressed when approaching imminent death [42]. Furthermore, neonatologists have been reported to first consider all life-sustaining treatment options before making an EOL decision, since making an EOL decision was less difficult when it was the only option to end the infant’s suffering [41]. However, bioethicists have argued it is important that parents understand and are enabled to assess *all* reasonable avenues of care, this includes EOL care, in order to provide informed consent [35]. Other authors have proposed to increase parental involvement with increasing prognostic uncertainty [20, 43]. Thus, providing parents with “all” options when certainty has been reached or death is imminent does not represent SDM [15]. It leaves room to speculate whether an uncertainty-based communication style cultivates or is a remnant of a paternalistic approach to decision-making for critically ill infants. Furthermore, such an approach might also reflect a practical hesitancy towards implementing SDM due to believed negative effects. Studies have shown that although HCPs in theory support SDM, still many physicians continue to be exceedingly worried about the possible burden placed on parents when actively involving them in the decision-making process [14, 26]. Such concerns have been criticised as overly protective in parental reports and observations [26, 44].

The presence of SDM guidelines and the explicit reference by neonatologists to shared approach in decision-making suggests SDM is a familiar concept in Swiss NICUs. Our findings illustrated that conversations were exclusively led by the neonatologists. However, nurses’ participation may improve continuity of care and provide parental support [45]. In addition, our study has shown that neonatologists rarely inquired about parents’ preferences and did not primarily give them full empowerment in decision-making. Similar to our findings, studies analysing the neonatal EOL decision-making process have found little evidence of neonatologists eliciting parents’ preferences [26, 36] and needs [42], which is one of the most important components in SDM along with medical evidence about reasonable treatment options [15, 46]. It has previously been described that -somewhat paradoxically- Swiss neonatologists and nurses support the idea of SDM but do not consider that parents can act in their child’s best interest [14, 25]. In fact, only a few HCPs would accept giving parents’ opinions more weight in ethical decisions regarding life-sustaining interventions [25]. Moreover, our findings identified that proactive parents were earlier and more involved in the decision-making process compared to parents who took a reactive approach in providing their opinion in decision-making. This outcome is supported by the findings of earlier studies in which parent-initiated EOL care discussion facilitated parental involvement in decision-making and increased the likelihood of different treatment options being presented by HCPs [26, 27]. In turn, Marlow et al. (2020) and Shaw et al. (2016) conversation analyses provide evidence on how neonatologists’ communication skills impact parental involvement in EOL decision-making. They observed that parents more frequently responded actively or freely asserted their preferences when presented with options than when given recommendations [47, 48]. Despite the extremely difficult situation parents find themselves in, it is suggested they may be credited with a more active role [26]. Although SDM is a well-known concept in Swiss NICUs, our findings provide evidence of the importance of further improving its implementation and practice among neonatal HCPs. Considering that most parents prefer an SDM approach [8, 19] and that parental non-involvement in decision-making can lead to feelings of powerlessness, anger, and grief among parents [8, 44] underscores the importance of continuing professional development and training in SDM.

All neonates retrospectively fulfilled the BAPM criteria for PPC and, in turn, most of infants died following the conversations. Hence, all infants were eligible in retrospect and parents could have been offered PPC information and services. Our results showed that palliative care was only discussed when parents took a proactive approach to decision-making or when the child’s death was imminent. We can only speculate about the reasons of neonatal HCPs for the lag in offering palliative care, e.g., of being trained to cure [36] or of being unfamiliar with the provision of palliative care services [13]. Notably, in a recent survey among Swiss NICUs, HCPs reported a lack of training and implementation of PPC guidelines and many expressed dissatisfaction with the provision of and need for training for PPC [13]. Early integration of PPC (teams) may enhance informed decision-making, navigate uncertainty, and provide grief support [35]. The Surprise Question [49] and the Paediatric Palliative Screening Scale [50] are potential strategies for HCPs to identify neonates who could benefit from palliative care early on and to prevent palliative care from being introduced late into the illness trajectory.

Despite the added richness of using a prospective design to describe the communication between HCPs and parents in neonatal EOL care, our study has several limitations. The study was restricted to two medical centres and included a limited number of participating HCPs and parents. Hence, we cannot generalise our findings as the results are not representative of all Swiss centres and HCPs. Future studies applying the same research design in other Swiss NICUs might provide a nationwide understanding. Furthermore, the data allowed us to report only what we recorded and reviewed based on documentation. Additional conversations between HCPs and parents have occurred during the infants’ hospitalization at the bedside, which could have provided additional information.

## Conclusion

Our results reflect how uncertainty inherent to these complex situations might impede on the process of shared decision-making. Strict adherence to the concept of certainty might constrain the value-based nature of such difficult decisions, thereby missing opportunities to include parental preferences and values. We further hold that communication, accepting and incorporating a certain degree of diagnostic and thus prognostic uncertainty, should entail providing all available options that respect the best interest of the neonate, and thus, allowing palliation to be discussed in eligible cases. Implementing tools such as the BAPM candidate conditions for PPC, the Surprise Question or the Paediatric Palliative Screening Scale should be further explored to help identify eligible cases and offer support to neonatal HCPs to initiate discussions on PPC with parents.

## Data Availability

The datasets used and/or analysed during the current study are available from the corresponding author on reasonable request.

## Abbreviations

BAPM: British Association of Perinatal Medicine
EOL: End-of-life
HCP: Healthcare professional
IVH: Intraventricular haemorrhage
NICU: Neonatal intensive care unit
n/a: not applicable
PPC: Perinatal palliative care
SDM: Shared decision-making
